# Effects of Purple Corn Anthocyanin on Blood Biochemical Indexes, Ruminal Fluid Fermentation, and Rumen Microbiota in Goats

**DOI:** 10.3389/fvets.2021.715710

**Published:** 2021-09-13

**Authors:** Xing-Zhou Tian, Jia-Xuan Li, Qing-Yuan Luo, Di Zhou, Qing-Meng Long, Xu Wang, Qi Lu, Gui-Lan Wen

**Affiliations:** ^1^College of Animal Science, Guizhou University, Guiyang, China; ^2^Institute of Animal Nutrition and Feed Science, Guizhou University, Guiyang, China; ^3^Key Laboratory of Animal Genetics, Breeding and Reproduction in the Plateau Mountainous Region, Ministry of Education, Guizhou University, Guiyang, China; ^4^Testing Center for Livestock and Poultry Germplasm, Guizhou Agricultural and Rural Affairs Office, Guiyang, China

**Keywords:** anthocyanins, purple corn, antioxidant capacity, rumen fermentation, microbial population

## Abstract

The objective of this study was to observe the effects of anthocyanin from purple corn on blood biochemical indexes, ruminal fluid fermentation parameters, and the microbial population in goats. A total of 18 Qianbei Ma wether kids (body weight, 21.38 ± 1.61 kg; mean ± standard deviation) were randomly assigned to three groups using a completely randomized design. The group diets were: (1) control, basal diet, (2) treatment 1 (LA), basal diet with 0.5-g/d purple corn pigment (PCP), and (3) treatment 2 (HA), basal diet with 1-g/d PCP. The results showed that supplementation of PCP anthocyanin increased (*P* < 0.05) crude protein and gross energy digestibilities compared to the control. Compared to the control group, the inclusion of anthocyanin-rich PCP led to significantly increased (*P* < 0.05) plasma reduced glutathione and peroxidase concentrations. Goats receiving PCP had increased (*P* < 0.05) ruminal fluid acetic acid and a higher ratio of acetate to propionate, while the propionic acid, butyric acid, valeric acid, isobutyric acid, and isovaleric acid levels had decreased (*P* < 0.05). There was no significant difference (*P* > 0.05) in ruminal fluid alpha bacterial diversity among the three groups. At the phylum level, the feeding of PCP had significant effect (*P* < 0.05) on the abundances of *Actinobacteriota, Proteobacteria, Elusimicrobiota*, WPS-2, and *Cyanobacteria*. At the genus level, HA group had lower (*P* < 0.05) *Prevotellaceae*_NK3B31_group abundance compared to the other groups. In addition, significant differences (*P* < 0.05) were also observed for the ruminal fluid *Eubacterium*_nodatum_group, *Amnipila, Ruminiclostridium*, U29-B03, unclassified_c_*Clostridia, Pyramidobacter, Anaeroplasma*, UCG-004, *Atopobium*, norank_f_norank_o_*Bradymonadales, Elusimicrobium*, norank_f_norank_o_norank_c_norank_p_WPS-2, norank_f_*Bacteroidales*_UCG-001, and norank_f_norank_o_*Gastranaerophilales* among all groups. Taken together, the inclusion of anthocyanin-rich PCP increased the antioxidant potential, improved rumen volatile fatty acids, and induced a shift in the structure and relative abundance of ruminal microbiota in growing goats.

## Introduction

Currently, plant secondary metabolites are being discussed as potential alternatives to conventional antibiotics in ruminant production (1). Anthocyanins are important secondary metabolites of plants, which provide color and serve as natural antioxidants (2). Purple corn has abundant anthocyanins, which is important sources of bioactivators, having many significant physiological functions and broad prospects for development and application (3). For example, purple corn anthocyanins could alleviate oxidative stress by improving plasma superoxide dismutase (SOD) concentration in lactating dairy cows (4).

The microorganism community in the rumen is important, as it can improve the metabolism of nutriments and maintain the stability of the inner rumen (5). Goats are more prone to oxidative stress during summer season which can be bound to cause rumen metabolic disorder (6). Anthocyanin-rich plants affected the immune response, perhaps inhibiting the inflammation processes by promoting microflora normalization and reducing the permeability of the gastrointestinal barrier (7). Yusuf et al. (8) revealed that phenol-rich plants could manipulate rumen metabolism by improving ruminal fluid populations of *Ruminococcus albus, Ruminococcus flavefaciens*, and *Fibrobacter succinogenes* in goats. Thus, anthocyanin can affect rumen fermentation parameters, as they can exert strong regulatory effects on ruminal microorganisms, improving the health of the gastrointestinal tract (9).

To our knowledge, however, studies on the effect of anthocyanins on goat rumen microorganisms *in vivo* are scarce. We hypothesized that goats receiving anthocyanins from purple corn would display effects on the ruminal fluid fermentation and microbial population, possibly providing an efficient natural additive for goat feedstuff. Accordingly, in the current study, 16S rRNA sequencing technology was applied to investigate the effects of anthocyanin-rich purple corn pigment (PCP) on the ruminal bacterial community, plasma biochemical indexes, and rumen fermentation parameters.

## Materials and Methods

### Purple Corn Anthocyanin and Animal

The PCP was purchased from Nanjing Herd Source Bio-technology Co., Ltd. (Nanjing, China). The anthocyanin composition of PCP was analyzed according to the method suggested by Tian et al. (10). The results showed that PCP had 1,974.75-μg/g cyanidin, 590.93-μg/g delphinidin, 45.33-μg/g pelargonidin, 7.93-μg/g petunidin, and 0.1-μg/g malvidin. Thus, the total anthocyanin content in PCP was 2,619.04-μg/g.

Heat stress has a negative impact on production performance, reproductive performance and immune performance of goats, and it can influence on rumen microorganisms. Hence, the feeding trial was conducted at a commercial goat farm (Xishui, Guizhou Province, China) during the summer season from 10 July to 24 September in 2020. Eighteen Qianbei Ma wether kids (body weight, 21.38 ± 1.61 kg; mean ± SD) were randomly assigned to three groups using a completely randomized design. The group diets were: (1) control, basal diet; (2) treatment 1 (LA), basal diet + 0.5-g/d purple corn pigment (PCP); and (3) treatment 2 (HA), basal diet + 1-g/d PCP. The preparation period was 14 d, and the formal experimental period was 60 d. The amount of PCP used in this research was determined based on the study by Tian et al. (11). The anthocyanin-rich PCP was mixed in concentrate, and then mixed with roughage to prepare total mixed rations (TMR). All goats were housed in clean pens with free access to water and were fed TMR (Table 1) and the nutrient requirements according to the NRC (12). Equal rations were provided twice daily at 08:30 and 16:30 for *ad libitum* intake and 10% refusals on an as-fed basis.

**Table 1:** Ingredients and nutrient composition of experimental diets (DM basis).

**Ingredients, %**	**Content**	**Chemical composition, %**	**Content**
Peanut vines	50.00	DM	90.17
White distiller's grains	10.00	CP	13.82
Soybean residues	10.00	GE, kJ/g	13.32
Green hay	9.30	NDF	43.54
Corn	14.00	ADF	29.23
Soybean meal	5.00	Hemicellulose	14.31
Mineral premix^a^	0.50	EE	2.27
Vitamin premix^b^	0.50	OM	91.53
NaCl	0.50	Ash	8.47
Limestone	0.20	Ca	1.04
Total	100.00	P	0.18

a *Vitamin premix was purchased from Guangzhou Everrich Animal Health Co., Ltd., (Guangzhou, China). It contained (per kg of premix): vitamin A, 4,000,000 IU; vitamin D, 600,000 IU; vitamin E, 25,000 mg; DL-methionine, 7,000 mg; L-lysine, 5,000 mg*.

b *Mineral premix was obtained from the Earth Animal Nutrition and Health Products Co., Ltd., (Chongqing, China). It contained (per kg of premix): Cu, 1,300 mg; Fe, 1,000 mg; Zn, 1,575 mg; Mn, 595 mg*.

*DM, dry matter; CP, crude protein; GE, gross energy; NDF, neutral detergent fiber; ADF, acid detergent fiber; EE, ether extract; OM, organic matter; Ca, calcium; P, phosphorus*.

### Chemical Composition

Approximately 500 g of basal diet was dried at 65°C in a vacuum oven for 72 h, and then ground and passed through a 1-mm sieve. The dry matter (DM, method 934.01), crude protein (CP, method 968.06), ether extract (EE, method 920.39), ash (method 942.05), calcium (Ca, method 927.02), and phosphorus (P, method 964.06) were measured as described in the AOAC (13). Neutral detergent fiber (NDF) and acid detergent fiber (ADF) were determined according to Van Soest et al. (14) using an FT 122 Fibertec^TM^ analyzer (Foss, Hillerød, Denmark). Organic matter (OM) and hemicellulose were obtained according to the following equations: OM = 100 – ash; hemicellulose = NDF – ADF. Gross energy (GE) was measured by a calorimeter (WGR-WR3, Changsha BENTE Instrument Co., Ltd., China). Each sample was run in triplicate.

### Temperature Humidity Index

The temperature humidity index (THI) was measured using a digital temperature-humidity recorder. The recorder was hung in the middle of the goat pens; this ensured that the hygrometer was well-ventilated and protected from sunlight and rain. The temperature and relative humidity were measured at 06:00, 14:00, and 20:00 every day throughout the experimental period. The THI was calculated according to the following equation (15):


$$\begin{array}{l} \text{THI}=0.8\times \text{AT}+\left(\text{RH}/100\right)\times \left(\text{AT}-14.4\right)+46.4, \end{array}$$

where AT is the temperature (°C), and RH is the relative humidity (%). When THI < 70, the goats were not heat stressed; 70 < THI < 75 indicated that the goats were under mild heat stress; 75 < THI < 78 indicated that the goats were under middle heat stress, and THI > 78 meant that the goats were under severe heat stress.

The temperature range was 15.00–27.33°C; the average temperature was 22.84°C; the humidity range was 53–98.33%; the average humidity was 78.49%, and the THI range was 58.93–77.29 with an average of 70.60.

### Apparent Digestibility

Fecal samples were collected on the last 5 d and apparent nutrient digestibility was determined by the acid-insoluble ash (AIA) method (16) using the following equation: apparent nutrient digestibility (%) =100% – (AIA in diet/AIA in fecal × nutrient in fecal/nutrient in diet) × 100. All of the fecal samples were oven-dried at 65°C for 72 h, ground and passed through a 1-mm sieve, and kept at 4°C until analysis.

### Blood Biochemical Indexes

A day before the experiment ended, 10-mL blood samples were taken at 0 and 3 h from the jugular vein using a vacuette tube with heparin sodium (Taizhou Qiujing Medical Instrument Co., Ltd., Jiangsu, China). The blood sample was transferred to a 1.5-mL tube after centrifugation (Allegra® X-30R Centrifuge, Beckman Coulter, USA) at 3,000 ×g for 15 min at 4°C, and was kept at −20°C until the blood biochemical indexes were analyzed.

The levels of glucose (GLU), urea nitrogen (UN), total protein (TP), albumin (Alb), reduced glutathione (GSH), peroxidase (POD), and 2,2-diphenyl-1-picrylhydrazyl (DPPH) scavenging capacity were determined using commercial kits (Jiancheng Bioengineering Institute, Nanjing, China). Product codes were F006-1-1, C013-2-1, A045-2, A028-1-1, A006-1-1, A048-3-1, and A153-1-1, respectively. All measurement procedures were strictly in accordance with the manufacturer's instructions.

### Rumen Fermentation Parameters

Approximately 30 mL of ruminal fluid was collected at the end of the experimental period. The ruminal fluid pH was determined immediately by a portable pH meter (pH 818, smart sensor, Guangdong, China). Ammonia nitrogen (NH_3_-N) was assayed according to the method of Bremner and Keeney (17).

About 100 μL of ruminal fluid was added to 900-μL deionized water, and the mixture was oscillated by a vortex meter (QL-866, Qilinbeier Instrument Manufacturing Co., Ltd., China) for 30 s. Next, 900 μL of diluted sample was added to 100 μL of 15% phosphoric acid and shaken vigorously; then, this mixture was added to 20 μL of 75-μg/mL internal standard (isohexanoic acid) solution and 280-μL ether for homogenization for 1 min. The supernatant was analyzed for individual volatile fatty acids (VFA) using a Thermo TRACE 1310-ISQ gas chromatography-mass spectrometer (GC-MS; Thermo Fisher Scientific, USA) after centrifugation at 12,000 × g at 4°C for 10 min (H1850R, Hunan Xiangyi Centrifuge Instrument Co., Ltd., China).

The GC conditions were as follows: separation of individual VFAs by an Agilent HP-INNOWAX capillary column (30 m × 0.25 mm × 0.25 μm); split stream sampling was applied; the injection volume was 1 μL, and the split ratio was 10:1; the injection port temperature was 250°C, the ionization temperature was 230°C, and the transmission line temperature was 250°C, The quadrupole temperature was 150°C. The temperature program was as follows: an initial temperature of 90°C, a 10°C/min rise to 120°C; and a 5°C/min rise to 150°C, followed by a 25°C/min rise to 250°C; this temperature was retained for 2 min. He gas was used as a carrier, and the flow rate of the carrier gas was 1.0 mL/min. The MS conditions were as follows: single ion monitoring scanning mode, ion energy and the energy of ionization was 70 eV.

### Total DNA Extraction and Detection

Total DNA was extracted from the ruminal fluid using a DNeasy® PowerSoil® Pro Kit (QIAGEN, USA). DNA purity and concentration were evaluated using NanoDrop2000 (Thermo Fisher Scientific, Waltham, Massachusetts, USA). Then, the integrity of the extracted DNA was assessed using an ImageQuant LAS 500 imager (GE Healthcare BioSciences) by electrophoresis on a 1% agarose gel, where the voltage was 5 V/cm and the run time was 20 min.

### PCR Amplification

Amplification of V3–V4 regions of the 16S rRNA gene for sequencing was conducted with the 338F and 806R primers (338F 5′-ACTCCTACGGGAGGCAGCAG-3′ and 806R 5′-GGACTACHVGGGTWTCTAAT-3′). TransStart Fastpfu DNA polymerase was used for the ABI GeneAmp® 9700PCR (Thermo Fisher Scientific, Waltham, Massachusetts, USA) with a 20-μL reaction volume: 4 μL of 5×FastPfu buffer, 2 μL of 2.5-mM dNTPs, 0.8 μL of forward primer (5 μM), 0.8 μL of reverse primer (5 μM), 0.4 μL of FastPfu polymerase, 0.2 μL of BSA, 10 ng of template DNA, and distilled water were added to a final volume of 20 μL. The PCR cycling conditions were as follows: initial denaturation at 95°C for 3 min, followed by 27 cycles of denaturation at 95°C for 30 s; annealing at 55°C for 30 s, and extension at 72°C for 45 s, with a final extension at 72°C for 10 min and at 10°C until the instrument stopped.

### Identification, Purification, and Quantification of PCR Products

Each sample was run in triplicate, and PCR products were mixed and analyzed by electrophoresis using 2% agarose gel. The PCR products were purified using an AxyPrep DNA Gel Extraction Kit (Axygen Biosciences, CA, USA). Each sample was in corresponding proportion and was stirred uniformly according to the sequencing quantity, and the PCR products were quantified using a Quantus^TM^ Fluorometer.

### Construction of a PE Library and Illumina Sequencing

The Miseq library was constructed using a NEXTFLEX® Rapid DNA-Seq Kit (Bio Scientific, USA), and the sequencing was performed by Miseq PE300 (Illumina, California, USA).

The quality control of the original sequences was performed by the Fastp software (https://github.com/OpenGene/fastp, version 0.20.0), and Flash software (http://www.cbcb.umd.edu/software/flash, version 1.2.7) was used for splicing. The procedure was follows: a 50-bp window was set to filter the bases when the tail mass was <20. The reads below 50 bp were filtered, and the reads containing N bases were removed. The paired reads were merged into one sequence according to the overlap relationship between PE reads, and the minimum overlap length was 10 bp. The maximum mismatch ratio of overlapping regions of a splicing sequence was 0.2; the number of mismatched barcodes was 0, and the maximum number of primer mismatches was set at 2.

The operational taxonomic unit (OTU) clustering was performed at 97% sequence identity using the UPARSE software (http://drive5.com/uparse/, version 7.1). Briefly, the non-repetitive sequences were extracted from the optimized sequences, and single sequences without repetition were removed. The OTU representative sequences were obtained by OTU clustering, and chimera removal was performed for nonrepetitive sequences (excluding single sequences) to generate an OTU table. All sequences were annotated by the Ribosomal Database Project classifier (http://rdp.cme.msu.edu/, version 2.2) and were compared with the Silva 16S rRNA database (v138), and the threshold was set at 70%.

### Statistical Analysis

The effect of PCP on plasma biochemical indexes and rumen fermentation parameters were performed by SAS 9.1.3 (SAS Institute, Cary, NC, USA) using one-way analysis of variance (ANOVA) with the following model: Y_*ij*_ = μ +τ_*i*_ + ε_*ij*_, where Y_*ij*_ is the *j* observation (*j* = 1–6) in the treatment *i* (*i* = control, LA, and HA), μ is the overall mean, τ_*i*_ is the effect of the treatment (denoted as an unknown parameter), and ε_*ij*_ is the random error with a mean of 0 and a variance of σ^2^.

The following data were analyzed on the free online Majorbio Cloud Platform (www.majorbio.com). Beta diversity distance measurements were performed employing the weighted and unweighted UniFrac distance matrix *via* QIIME (V1.8.0), and visualized with the principal coordinate analysis (PCoA). Alpha diversity (Sobs, Shannon, Simpson, Ace, Chao, and Coverage) was measured by the Mother software (version v.1.30.2 http://www.mothur.org/wiki/Schloss_SOP#Alpha_diversity). The effect of PCP on rumen bacteria at the phylum and genus levels were applied using a non-parametric test (Kruskal–Wallis test) in this study. Each goat was considered an experimental unit. The significance level was set at *P* < 0.05.

## Results

### Apparent Digestibility

The dry matter intake of the control, LA and HA groups was 701.84, 714.67, and 720.62 g/d, respectively. No difference (*P* > 0.05) in average daily gain (56.25, 54.06, and 58.08 g, respectively) was noted among all groups. The apparent digestibilities of DM, OM, EE, NDF, ADF, Ca, and P were not affected (*P* > 0.05) by anthocyanin, but GE and CP digestibilities were significantly higher (*P* < 0.05) in the PCP groups than that in the control (Table 2).

**Table 2:** Effect of purple corn anthocyanin on apparent digestibility of dairy goats^a^.

**Items^**b**^, %**	**Group** ^**c**^			**SEM**	***P*-value**
	**Control**	**LA**	**HA**		
DM	85.65	87.41	85.52	0.6928	0.1788
OM	73.45	76.98	72.59	1.3725	0.1331
GE	73.40^c^	77.28^a^	74.93^b^	0.0819	<0.0001
CP	69.92^b^	73.67^a^	71.24^a^^b^	0.7483	0.0318
EE	75.36	71.49	69.29	3.3277	0.4723
NDF	49.61	51.39	43.81	2.2568	0.1201
ADF	42.30	45.99	42.60	1.6063	0.2732
Ca	53.19	53.19	58.46	2.7940	0.3677
P	57.13	57.21	59.70	1.2390	0.3201

a *Different letters within a row are significantly different (P < 0.05)*.

b *Values represent the mean of six replicates (n = 6)*.

c *LA = basal diet + 0.5-g/d PCP; HA = basal diet + 1-g/d PCP; SEM, standard error of mean*.

*DMI, dry matter; OM, organic matter; GE, gross energy; CP, crude protein; EE, ether extract; NDF, neutral detergent fiber; ADF, acid detergent fiber; Ca, calcium; P, phosphorus. Different letters within a row denote significant differences (P < 0.05)*.

### Plasma Biochemical Indexes

There were no significant differences (*P* > 0.05) for GLU, UN, TP, Alb, or DPPH scavenging activity among the groups (Table 3). However, goats receiving PCP had significantly increased (*P* < 0.05) GSH and POD concentrations relative to the control group.

**Table 3:** Effect of purple corn anthocyanin on blood biochemical indexes of dairy goats^a^.

**Items^**b**^**	**Group** ^**c**^			**SEM**	***P*-value**
	**Control**	**LA**	**HA**		
GLU, mmol/L	5.69	5.22	5.24	0.264	0.4948
UN, mmol/L	5.44	5.37	5.16	0.279	0.7561
TP, gprot/L	4.09	4.36	4.26	0.100	0.1787
Alb, g/L	23.65	23.86	22.64	1.164	0.7425
GSH, mg/L	6.19^b^	8.81^a^	7.51^a^^b^	0.617	0.0387
POD, U/mg	1.28^b^	3.49^a^	3.38^a^	0.402	0.0024
DPPH scavenging activity, %	52.21	51.43	50.59	0.620	0.1957

a *Different letters within a row denote significant differences (P < 0.05)*.

b *Values represent the mean of six replicates (n = 6)*.

c *LA = basal diet + 0.5-g/d PCP; HA = basal diet + 1-g/d PCP; SEM, standard error of mean*.

*GLU, glucose; UN, urea nitrogen; TP, total protein; Alb, albumin; GSH, reduced glutathione; POD, peroxidase; DPPH, 2,2-diphenyl-1-picrylhydrazyl. Different letters within a row denote significant differences (P < 0.05)*.

### Rumen Fermentation Parameters

No significant differences (*P* > 0.05) were observed for ruminal fluid pH, NH_3_-N, or total VFA among the three groups (Table 4). Compared with the control group, the inclusion of PCP increased (*P* < 0.05) ruminal fluid acetic acid and the ratio of acetate to propionate, whereas it decreased (*P* < 0.05) propionic acid, butyric acid, valeric acid, isobutyric acid, and isovaleric acid levels.

**Table 4:** Effect of purple corn anthocyanin on ruminal fluid fermentation parameters of dairy goats^a^.

**Item^**b**^**	**Group** ^**c**^			**SEM**	***P*-value**
	**Control**	**LA**	**HA**		
pH	7.22	7.15	7.19	0.052	0.1471
NH_3_-N, mg/dL	11.67	11.34	11.80	0.443	0.7550
Total VFA, mM	107.22	100.99	102.99	4.9057	0.6909
**Individual VFA, molar % of total VFA**					
Acetic acid	53.789^b^	63.543^a^	61.791^a^	1.049	<0.0001
Propionic acid	19.973^a^	15.090^b^	17.159^b^	0.718	0.0009
Butyric acid	13.958^a^	11.467^b^	12.392^b^	0.500	0.0101
Valeric acid	2.472^a^	1.711^b^	1.738^b^	0.060	<0.0001
Caproic acid	0.057^a^	0.030^b^	0.058^a^	0.002	<0.0001
Isobutyric acid	4.891^a^	4.137^b^	3.459^c^	0.164	<0.0001
Isovaleric acid	4.878^a^	4.032^b^	3.406^c^	0.171	<0.0001
Ratio of acetate to propionate	2.724^c^	4.274^a^	3.608^b^	0.169	<0.0001

a *Different letters within a row denote significant differences (P < 0.05)*.

b *Values represent the mean of six replicates (n = 6)*.

c *LA = basal diet + 0.5-g/d PCP; HA = basal diet + 1-g/d PCP; SEM, standard error of mean. NH_3_-N, ammonia nitrogen; VFA, volatile fatty acid. Different letters within a row denote significant differences (P < 0.05)*.

### Alpha Diversity Analysis

A total of 1,023,437 high-quality sequences were obtained; the effective bases number was 426,450,118 bp, and the average length was 416 nt from 18 samples. For OTU cluster analysis, a total of 1,604 OTUs were obtained, and these were classified into 18 phyla, 34 classes, 68 orders, 112 families, 215 genera, and 424 species. As shown in Table 5, no differences (*P* > 0.05) were observed for Sobs, Shannon, Simpson, Ace, Chao, or Coverage indexes among the three groups.

**Table 5:** Analysis of α diversity index in this study.

**Item^**a**^**	**Group** ^**b**^			**SEM**	***P*-value**
	**Control**	**LA**	**HA**		
Sobs	1,132.00	1,122.17	1,162.17	22.218	0.4351
Shannon	5.35	5.36	5.34	0.060	0.9830
Simpson	0.017	0.015	0.018	0.002	0.6139
Ace	1,300.86	1,283.14	1,317.50	25.481	0.6431
Chao	1,319.97	1,292.02	1,328.57	27.280	0.6217
Coverage	0.9925	0.9928	0.9927	0.0002	0.6074

a *Values represent the mean of six replicates (n = 6)*.

b *LA = basal diet + 0.5-g/d PCP; HA = basal diet + 1-g/d PCP; SEM, standard error of mean*.

**Figure 1:**
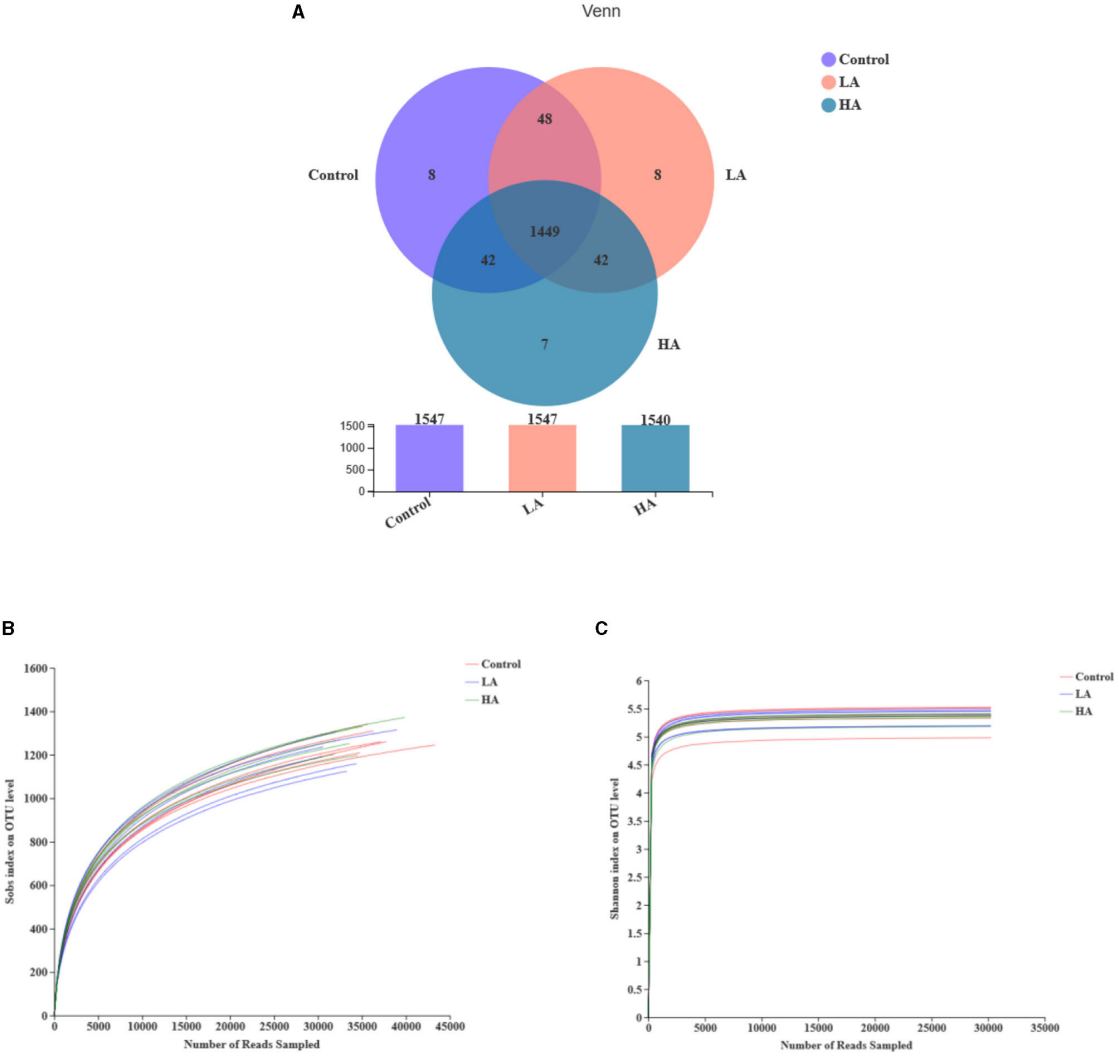
Effect of anthocyanin-rich purple corn on ruminal fluid bacterial diversity. **(A)** The Venn diagram. **(B)** The rarefaction curves. **(C)** The Shannon curves. LA = basal diet + 0.5-g/d PCP; HA = basal diet + 1-g/d PCP.

The control, LA, and HA groups had 1,547, 1,547, and 1,540 OTUs, respectively. In addition, 1,449 identical OTUs were noted among the three groups, and 8, 8, and 7 individual OUTs were detected for the control, LA, and HA groups, respectively (Figure 1A). The rarefaction curves tended to be flat with the increase of sequence number, indicating that the sequencing data were reasonable (Figure 1B). The Shannon curves had reached saturation, meeting the detection requirements (Figure 1C).

### Beta Diversity Analysis

**Figure 2:**
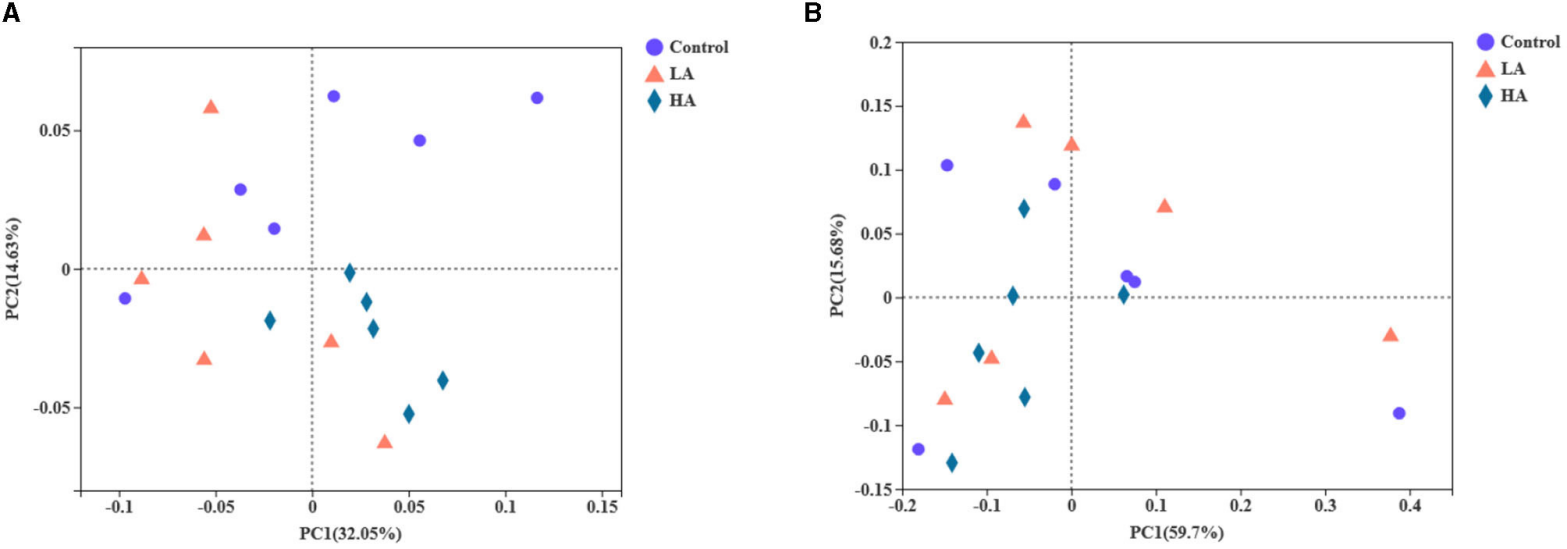
Effect of anthocyanin-rich purple corn on ruminal fluid beta diversity analysis. **(A)** PCoA based on unweighted unifrac distance. **(B)** PCoA based on weighted unifrac distance. LA = basal diet + 0.5-g/d PCP; HA = basal diet + 1-g/d PCP.

The PCoA was noted based on unweighted unifrac and weighted unifrac distances, and the contribution values of principal component (PC) 1 and PC2 for sample differences were 32.05, 14.63 and 59.70, 15.68%, respectively (Figures 2A,B). The three groups were not clearly separated, and there was overlapping. Unweighted unifrac was analyzed for the existence and evolutionary relationships of species, which indicated that the control and LA groups had greater species abundance than the HA group. Weighted unifrac mainly integrated the analysis of evolutionary relationships and relative abundance of major species, to identify similarities and differences in microbiota.

### Rumen Bacteria at the Phylum Level

**Figure 3:**
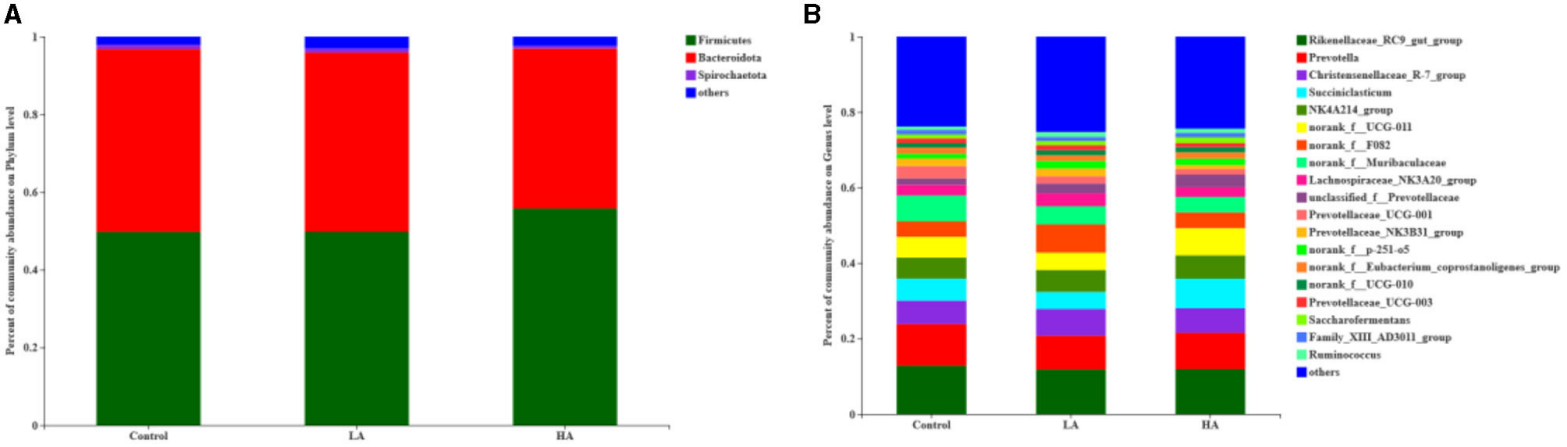
Effect of anthocyanin-rich purple corn on rumen dominant bacteria (>1%; as a percentage of the total sequence). **(A)** Phylum level. **(B)** Genus level. LA = basal diet + 0.5-g/d PCP; HA = basal diet + 1-g/d PCP.

**Figure 4:**
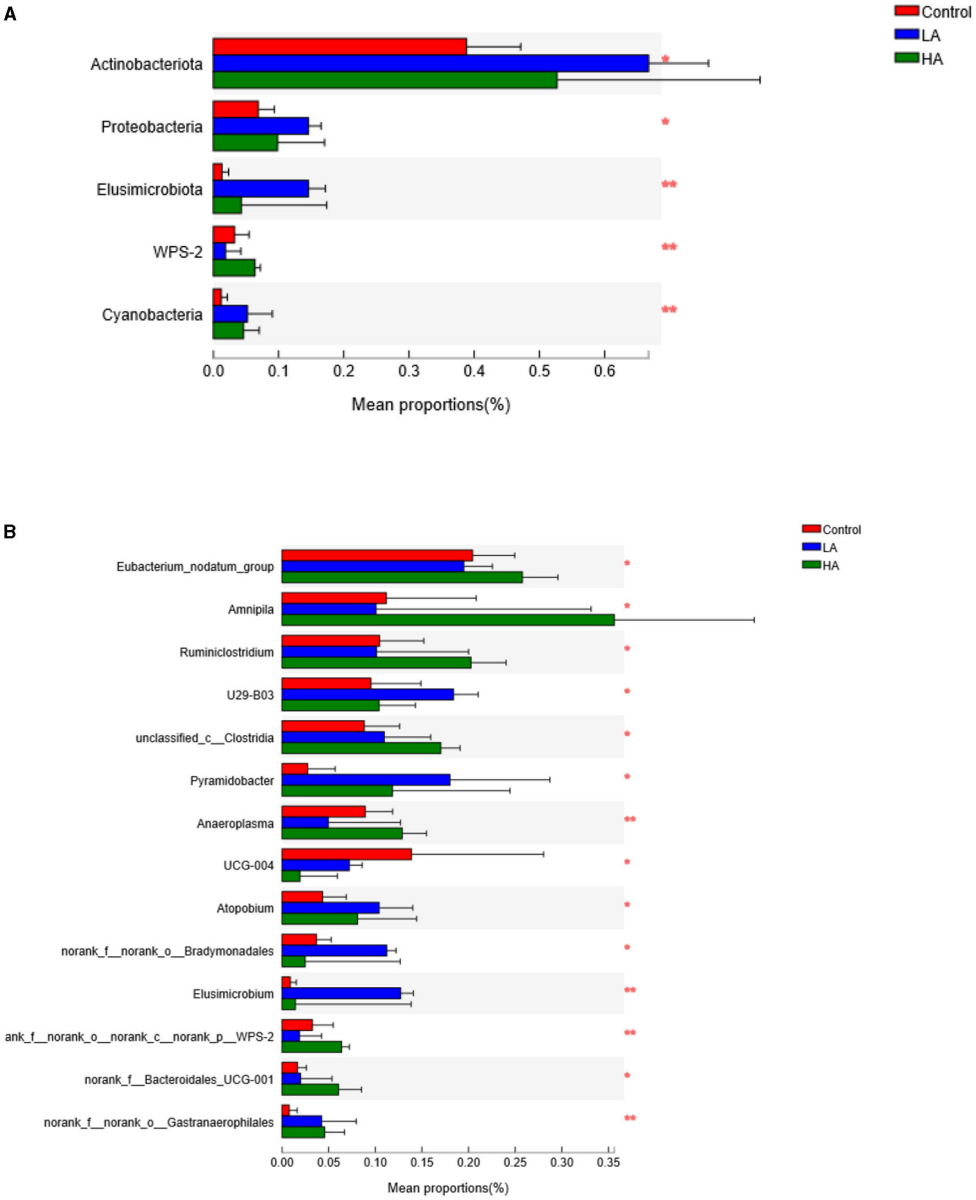
Analysis of the significant differences of microbiota composition (<1%). **(A)** Phylum level. **(B)** Genus level. ^*^P < 0.05, ^**^P < 0.01. LA = basal diet + 0.5-g/d PCP; HA = basal diet + 1-g/d PCP.

For the ruminal microbiota with relative abundance >1%, the addition of PCP had no effect (*P* > 0.05) on the *Firmicutes, Bacteroidota*, or *Spirochaetota* in the rumen (Table 6; Figure 3A). However, for the ruminal microbiota with relative abundance <1%, *Actinobacteriota, Proteobacteria, Elusimicrobiota*, WPS-2, and *Cyanobacteria* had significant differences (*P* < 0.05) among all groups (Figure 4A).

**Table 6:** Effect of purple corn anthocyanin on the relative abundance of rumen dominant microflora at the phylum level (>1%).

**Item^**a**^**	**Group** ^**b**^			**SEM**	***P*-value**
	**Control**	**LA**	**HA**		
*Firmicutes*	49.79	49.82	55.76	3.653	0.3722
*Bacteroidota*	47.00	46.11	41.16	3.458	0.3594
*Spirochaetota*	1.14	1.06	0.70	0.301	0.3993

a *Values represent the mean of six replicates (n = 6)*.

b *LA = basal diet + 0.5-g/d PCP; HA = basal diet + 1-g/d PCP; SEM, standard error of mean*.

### Rumen Bacteria at Genus Level

The feeding of goats with anthocyanin from PCP had no effect (*P* > 0.05) on the most of rumen bacteria with relative abundance >1% at the genus level, except for the *Prevotellaceae*_NK3B31_group (*P* < 0.05, Table 7; Figure 3B). The HA group showed lower (*P* < 0.05) *Prevotellaceae*_NK3B31_group abundance relative to the other groups during the entire period. In addition, for the rumen bacteria with relative abundance <1%, the feeding of goats with anthocyanin-rich purple corn had a significant effect (*P* < 0.05) on *Eubacterium*_nodatum_group, *Amnipila, Ruminiclostridium*, U29-B03, unclassified_c_*Clostridia, Pyramidobacter, Anaeroplasma*, UCG-004, *Atopobium*, norank_f_norank_o_*Bradymonadales, Elusimicrobium*, norank_f_norank_o_norank_c_norank_p_WPS-2, norank_f_*Bacteroidales*_UCG-001, and norank_f_norank_o_*Gastranaerophilales* (Figure 4B).

**Table 7:** Effect of purple corn anthocyanin on the relative abundance of rumen dominant microflora at the genus level (>1%).

**Item^**a**^**	**Group** ^**b**^			**SEM**	***P*-value**
	**Control**	**LA**	**HA**		
*Rikenellaceae*_RC9_gut_group	12.83	11.87	11.91	1.628	0.8342
*Prevotella*	11.02	8.90	9.62	1.872	0.7961
*Christensenellaceae*_R-7_group	6.22	7.05	6.50	0.498	0.3594
*Succiniclasticum*	5.85	4.55	7.87	1.823	0.3235
NK4A214_group	5.53	5.79	6.13	0.563	0.7777
norank_f__UCG-011	5.50	4.61	7.24	0.890	0.1911
norank_f__F082	4.12	7.50	4.18	1.265	0.1190
norank_f__*Muribaculaceae*	6.86	4.80	4.11	2.019	0.5287
*Lachnospiraceae*_NK3A20_group	2.80	3.44	2.54	0.287	0.1720
unclassified_f__*Prevotellaceae*	1.75	2.60	3.41	0.782	0.1492
*Prevotellaceae*_UCG-001	3.23	1.93	1.47	0.769	0.3722
*Prevotellaceae*_NK3B31_group	2.03^a^	2.06^a^	0.97^b^	0.306	0.0380
norank_f__p-251-o5	1.22	1.98	1.72	0.474	0.5195
norank_f__*Eubacterium*_coprostanoligenes_group	1.68	1.49	1.69	0.207	0.8342
norank_f__UCG-010	1.14	1.34	1.38	0.144	0.4703
*Prevotellaceae*_UCG-003	1.29	1.33	1.05	0.315	0.9599
*Saccharofermentans*	1.02	1.09	1.53	0.205	0.1443
Family_XIII_AD3011_group	1.25	1.12	1.20	0.156	0.7777
*Ruminococcus*	0.86	1.29	1.11	0.138	0.1254

a *Values represent the mean of six replicates (n = 6)*.

b *LA = basal diet + 0.5-g/d PCP; HA = basal diet + 1-g/d PCP; SEM, standard error of mean. ^a,b^Different letters within a row denote significant differences (P < 0.05)*.

## Discussion

The addition of PCP could improve GE digestibility in the present study. This is may be because the sugar structure in anthocyanin might be involved in digestion and metabolism (18), and whether this is true or is a chance finding requires further study. In addition, anthocyanins can bind to dietary proteins and reduce rumen fermentation, thereby increasing nitrogen utilization (19). As expected, we found that feeding PCP could increase CP digestibility, which may be related to the high anthocyanin content in PCP. Consistent with our results, Nudda et al. (20) demonstrated that ewes receiving polyphenol-rich plants exhibited increased nitrogen utilization and CP digestibility.

The heat stress is the most important factor affecting the production and reproductive potential of small ruminants (21). This is mainly owing to the heat stress results from an imbalance between the generation of oxygen derive radicals and the organism's antioxidant potential (22). Anthocyanins are potent antioxidants that regulate peroxidation reactions and control free radical production in goat body (23). The previous studies revealed that anthocyanins can donate phenolic hydrogen atoms and capture free radicals, thereby stabilizing the compounds through various resonance forms once they are oxidized (24, 25). Therefore, anthocyanin groups showed higher levels of GSH and POD, indicating that anthocyanins have the ability to improve antioxidant activity and alleviate heat stress, and they can be used as new functional feed additives for ruminants. This may be because anthocyanins could impact the microbes in the gastrointestinal tract through various antioxidant signaling pathways, such as the ROS/JNK and NF-κB/BACE1 pathways (26). Additionally, the bacteria in the gastrointestinal tract participate in the activation and absorption of anthocyanins, enhance intestinal barrier function, increase mucus secretion, and regulate intestinal immune response through cytokine stimulation (27, 28). Our results are consistent with those of Matsuba et al. (29), who reported that the inclusion of anthocyanin-rich purple corn (*Zea mays* L.) silage could increase the antioxidant potential through enhanced blood SOD concentration in lactating cows. Similarly, Tian (30) found that feeding anthocyanin-rich purple corn stover silage could enhance the antioxidant potential by improving the expression of related antioxidant genes in the mammary gland.

In the present study, there was no significant difference in ruminal fluid pH among the three groups, meaning that PCP could maintain an appropriate acid-base status without inducing rumen acidosis. The VFAs are the main sources of energy for metabolism in ruminants. Anthocyanins could increase the formation of VFAs by regulating intestinal flora (31). Purba et al. (32) demonstrated that flavonoid-rich plants could change rumen fermentation type in goats. This may be because the hydroxyl groups in anthocyanins are the main groups responsible for inhibitory activity and, as such, may break bacterial cell membranes (33). Hence, the feeding of PCP altered the fermentation mode of VFAs. The potential mechanism might involve anthocyanins reducing gastrointestinal oxygen tension, regulating the bacterial population, and VFA production (34). In addition, anthocyanin could bind with proteins in the bacterial cell walls and cell membranes and change in the morphology of extracellular enzymes, thereby affecting bacterial activities (19, 35). Furthermore, we found that the inclusion of PCP reduced the propionate concentration, suggesting that PCP could inhibit CH_4_ production due to competition for hydrogen. However, this needs further investigation in future studies. Consistent with our results, Hosoda et al. (36) demonstrated that ruminal fluid acetic acid tended to increase after sheep received anthocyanin-rich purple rice. However, a previous study demonstrated that consuming anthocyanin-rich plants did not affect ruminal fluid VFA content (37). Interestingly, anthocyanin-rich plants are not easily degraded by rumen fluid (38), but purple extract is easily degraded by rumen fluid (25). Moreover, the fermentation substrate, rumen environment, and microbial population are the important factors affecting the type of VFA in the rumen (39). Hence, this difference may be caused by fermentation products, anthocyanin sources, and animal physiological stages.

A previous study demonstrated that when the body is under oxidative stress, this can change the type and content of microorganisms in the gastrointestinal tract (26). Chikwanha et al. (40), who showed that polyphenol-rich grape pomace could improve rumen microbial diversity, evenness, and richness in lambs in *in vitro* fermentation experiments. Wang et al. (41) demonstrated that supplementation of phenolic-rich inulin in the diet could increase the relative abundance of commensal microbiota in dairy cows. It was likely that the microorganisms could improve anthocyanin bioavailability in the gastrointestinal tract, enhancing the gut antioxidant capacity (42). In addition, anthocyanins have strong antioxidant activity, which can protect the body from peroxidation damage, thus improving rumen microflora. As a consequence, anthocyanins can reduce heat stress and change the rumen microflora, thus improving rumen health. In the present study, goats were under mild heat stress, meaning that rumen microbes would be disturbed. Anthocyanins as gut modifiers could promote the proliferation of healthy anaerobic bacteria and inhibit pathogenic bacteria (43). Therefore, we found the feeding of anthocyanin could increase various bacteria abundances. Consistent with our results, Bryszak et al. (44) showed that anthocyanin-rich berry seed residues could increase ruminal fluid total bacteria in a batch culture system. Moreover, Sun et al. (45) showed that dairy cows receiving anthocyanin-rich plants had increased relative abundance of ruminal fluid *Marvinbryantia, Acetitomaculum, Ruminococcus gauvreauii, Eubacterium coprostanoligenes, Selenomonas*_1, *Pseudoscardovia*, norank_f_*Muribaculaceae*, and *Sharpea*.

Anthocyanins are formed from anthocyanidin with one or more glucose, rhamnose, galactose, xylose, and arabinose units through glycosidic bonds under natural conditions (46). Additionally, the hydrolysis of anthocyanins occurs by cleavage of the sugar moiety and formation of aglycon, and this is degraded into simple phenolic acids by the activities of bacterial enzymes in the gastrointestinal tract (47). These previous reports lead us to presume that the sugars in anthocyanins might be broken down in the rumen and alter rumen microflora, and they were capable of using anthocyanin as a source of carbon, but this hypothesis needs further verification. In addition, athocyanins have antibacterial activity and inhibit the growth of a variety of pathogens due to intracellular interactions (48). Gutierrez-Banuelos et al. (49) revealed that phenolic-rich plants had antimicrobial effects in feedlot cattle. We found the HA group had decreased relative abundance of ruminal fluid *Prevotellaceae*_NK3B31_group compared to the control group, suggesting that high anthocyanin content inhibited microorganisms in the rumen. Of interest, feeding anthocyanins had less effect on rumen microbes with relative abundances of more than 1%, whereas it had a greater effect on rumen microorganisms with relative abundance <1%. The reasons may be as follows: (1) The bioavailability of anthocyanins *in vivo* is very low; the high relative abundance of microbes may need more anthocyanin; (2) Different microbes have different ways of absorbing anthocyanins; and (3) The relative abundance of some microbes increased, indirectly affecting the growth of other microbes.

## Conclusions

The present study indicated that anthocyanin-rich PCP could be used as a functional feed additive, as suggested by (1) the benefits to plasma antioxidant enhancement; (2) changes in the rumen fermentation type and improves VFAs; (3) anthocyanin from purple corn affecting rumen microflora by regulating the relative abundance of rumen microbes and improving rumen microbial diversity. Further feeding trials in ruminants are needed to determine the mechanism by which anthocyanins affect rumen microbes.

## Data Availability Statement

The datasets presented in this study can be found in online repositories. The names of the repository/repositories and accession number(s) can be found below: https://www.ncbi.nlm.nih.gov/, PRJNA732572.

## Ethics Statement

The protocol for this experiment was approved by the Inspection Form for the Guizhou University, Experimental Animal Ethics (EAE-GZU-2020-7009).

## Author Contributions

X-ZT: resources, data curation, and writing—original draft preparation. J-XL: investigation, revising, and editing. Q-YL: revising and editing. XW: investigation. DZ: validation. Q-ML: supervision. G-LW: data curation, software, and conceptualization. QL: conceptualization, methodology, writing—reviewing, and editing. All authors drafted and approved the final version of the manuscript.

## Funding

This work was supported by the Start-up Funds of Guizhou University (2016-76; 2019-26), the Cultivating Project of Guizhou University (2019-33), the Science and Technology Project of Guizhou Province (Qiankehe foundation-ZK [2021] General 164), the State Key Laboratory of Animal Nutrition (2004DA125184F1914), the National Natural Science Foundation of China (32060794), and the Central Guidance to Local Funds, Grant (QKZY-2018-4015).

## Conflict of Interest

The authors declare that the research was conducted in the absence of any commercial or financial relationships that could be construed as a potential conflict of interest.

## Publisher's Note

All claims expressed in this article are solely those of the authors and do not necessarily represent those of their affiliated organizations, or those of the publisher, the editors and the reviewers. Any product that may be evaluated in this article, or claim that may be made by its manufacturer, is not guaranteed or endorsed by the publisher.

## References

[1] Ku-Vera JC Jimenez-Ocampo R Valencia-Salazar SS Montoya-Flores MD Molina-Botero IC Arango J. Role of secondary plant metabolites on enteric methane mitigation in ruminants. Front Vet Sci. (2020) 7:784. 10.3389/fvets.2020.00584 33195495 PMC7481446

[2] Bagchi D Sen CK Bagchi M Atalay M. Anti-angiogenic, antioxidant, and anti-carcinogenic properties of a novel anthocyanin-rich berry extract formula. Biochemistry. (2004) 69:75–80. 10.1023/B:BIRY.0000016355.19999.93 14972022

[3] Colombo R Ferron L Papetti A. Colored corn: an up-date on metabolites extraction, health implication, and potential use. Molecules. (2021) 26:199. 10.3390/molecules26010199 33401767 PMC7796034

[4] Hosoda K Eruden B Matsuyama H Shioya S. Effect of anthocyanin-rich corn silage on digestibility, milk production and plasma enzyme activities in lactating dairy cows. Anim Sci J. (2012) 83:453–59. 10.1111/j.1740-0929.2011.00981.x 22694328

[5] Langda S Zhang C Zhang K Gui B Ji D Deji C. Diversity and composition of rumen bacteria, fungi, and protozoa in goats and sheep living in the same high-altitude pasture. Animals. (2020) 10:186. 10.3390/ani10020186 31978949 PMC7070549

[6] Paul A Dangi SS Gupta M Singh J Sarkar M. Expression of TLR genes in black bengal goat (*Capra hircus*) during different seasons. Small Ruminant Res. (2015) 124:17–23. 10.1016/j.smallrumres.2015.01.011

[7] Pieszka M Gogol P Pietras M Pieszka M. Valuable components of dried pomaces of chokeberry, black currant, strawberry, apple and carrot as a source of natural antioxidants and nutraceuticals in the animal diet. Ann Anim Sci. (2015) 15:475–91. 10.2478/aoas-2014-0072

[8] Yusuf AL Adeyemi KD Samsudin AA Goh YM Alimon AR Sazili AQ. Effects of dietary supplementation of leaves and whole plant of andrographis paniculata on rumen fermentation, fatty acid composition and microbiota in goats. BMC Vet Res. (2017) 13:349. 10.1186/s12917-017-1223-0 29178910 PMC5701315

[9] Pereira S. Protective Role of Anthocyanins on Intestinal Inflammation in Comparison With 5-Aminosalicylic Acid: In vitro and In vivo Approaches (Dissertation/Ph.D thesis). University of Coimbra, Coimbra (2018).

[10] Tian XZ Lu Q Paengkoum P Paengkoum S. Short communication: effect of purple corn pigment on change of anthocyanin composition and unsaturated fatty acids during milk storage. J Dairy Sci. (2020) 103:7808–12. 10.3168/jds.2020-18409 32684465

[11] Tian XZ Xin HL Paengkoum P Paengkoum S Ban C Thongpea S. Effects of anthocyanin-rich purple corn (*Zea mays* L.) stover silage on nutrient utilization, rumen fermentation, plasma antioxidant capacity, and mammary gland gene expression in dairy goats. J Anim Sci. (2019) 97:1384–97. 10.1093/jas/sky477 30576545 PMC6396244

[12] National Research Council. Nutrient Requirements of Goats: Angora, Dairy, and Meat Goats in Temperate and Tropical Countries. Washington, DC: The National Academies Press (1981).

[13] Association of Official Analytical Chemists. Association of Official Analytical Chemists Official Methods of Analysis, 18th Edn. Washington, DC: AOAC (2005).

[14] Van Soest PV Robertson JB Lewis BA. Methods for dietary fiber, neutral detergent fiber, and nonstarch polysaccharides in relation to animal nutrition. J Dairy Sci. (1991) 74:3583–97. 10.3168/jds.S0022-0302(91)78551-2 1660498

[15] Minka NS Ayo JO. Assessment of thermal load on transported goats administered with ascorbic acid during the hot-dry conditions. Int J Biometeorol. (2012) 56:333–41. 10.1007/s00484-011-0437-2 21544699

[16] Taniguchi K Yamatani Y Otani I. Acid-insoluble ash as an indicator for determining the feed consumption and the digestibility in the lactating dairy cow. J Jpn J Zootech Sci. (1986) 5:438–41. 10.2508/chikusan.57.438

[17] Bremner JM Keeney DR. Steam distillation methods for determination of ammonium, nitrate and nitrite. Anal Chim Acta. (1965) 32:485–95. 10.1016/S0003-2670(00)88973-4

[18] Ribnicky DM Roopchand DE Oren A Grace M Poulev A Lila MA. Effects of a high fat meal matrix and protein complexation on the bioaccessibility of blueberry anthocyanins using the TNO gastrointestinal model (TIM-1). Food Chem. (2014) 142:349–57. 10.1016/j.foodchem.2013.07.073 24001852 PMC4072317

[19] Correddu F Lunesu MF Buffa G Atzori AS Nudda A Battacone G. Can agro-industrial by-products rich in polyphenols be advantageously used in the feeding and nutrition of dairy small ruminants? Animals. (2020) 10:131. 10.3390/ani10010131 31947543 PMC7022336

[20] Nudda A Correddu F Atzori AS Marzano A Battacone G Nicolussi P. Whole exhausted berries of *Myrtus communis* L. supplied to dairy ewes: effects on milk production traits and blood metabolites. Small Ruminant Res. (2017) 155:33–38. 10.1016/j.smallrumres.2017.08.020

[21] Sejian V Bagath M Krishnan G Rashamol VP Pragna P Devaraj C. Genes for resilience to heat stress in small ruminants: a review. Small Ruminant Res. (2019) 173:42–53. 10.1016/j.smallrumres.2019.02.009

[22] Sivakumar A Singh G Varshney VP. Antioxidants supplementation on acid base balance during heat stress in goats. Asian-Austral J Anim. (2010) 23:1462–68. 10.5713/ajas.2010.90471

[23] Tian X Lu Q Zhao S Li J Luo Q Wang X. Purple corn anthocyanin affects lipid mechanism, flavor compound profiles, and related gene expression of Longissimus Thoracis et Lumborum muscle in goats. Animals. (2021) 11:2407. 10.3390/ani11082407 34438864 PMC8388639

[24] Tian XZ Paengkoum P Paengkoum S Chumpawadee S Ban C Thongpea S. Short communication: purple corn (*Zea mays* L.) stover silage with abundant anthocyanins transferring anthocyanin composition to the milk and increasing antioxidant status of lactating dairy goats. J Dairy Sci. (2019) 102:413–18. 10.3168/jds.2018-15423 30415857

[25] Leatherwood WL. The Effect of Anthocyanins from Purple-Fleshed Sweetpotato on In vitro Fermentation by Rumen Microbial Cultures (dissertation/master's thesis). North Carolina State University, Raleigh (2014).

[26] Khan MS Ikram M Park JS Park TJ Kim MO. Gut microbiota, its role in induction of Alzheimer's disease pathology, and possible therapeutic interventions: special focus on anthocyanins. Cells. (2020) 9:853. 10.3390/cells9040853 32244729 PMC7226756

[27] Eker ME Aaby K Budic-Leto I Brnčić SR El SN Karakaya S. A Review of factors affecting anthocyanin bioavailability: possible implications for the inter-individual variability. Foods. (2020) 9:2. 10.3390/foods9010002 31861362 PMC7023094

[28] Li S Wu B Fu W Reddivari L. The anti-inflammatory effects of dietary anthocyanins against ulcerative colitis. Int J Mol Sci. (2019) 20:2588. 10.3390/ijms20102588 31137777 PMC6567294

[29] Matsuba T Kubozono H Saegusa A Obata K Gotoh K Miki K. Short communication: effects of feeding purple corn (*Zea mays* L.) silage on productivity and blood superoxide dismutase concentration in lactating cows. J Dairy Sci. (2019) 102:7179–82. 10.3168/jds.2019-16353 31178175

[30] Tian XZ. Effects of Anthocyanin-Rich Purple Corn (Zea mays L.) Stover Silage on Antioxidant Activities in Dairy Goats (Dissertation/Ph.D thesis). Suranaree University of Technology, Nakhon Ratchasima (2018).

[31] Li J Wu T Li N Wang X Chen G Lyu X. Bilberry anthocyanin extract promotes intestinal barrier function and inhibits digestive enzyme activity by regulating the gut microbiota in aging rats. Food Funct. (2018) 10:333–43. 10.1039/C8FO01962B 30575836

[32] Purba R Paengkoum S Yuangklang C Paengkoum P. Flavonoids and their aromatic derivatives in Piper betle powder promote *in vitro* methane mitigation in a variety of diets. Cienc Agrotec. (2020) 44:e012420. 10.1590/1413-7054202044012420

[33] Mattos GN Tonon RV Furtado A Cabral L. Grape by-product extracts against microbial proliferation and lipid oxidation: a review. J Sci Food Agr. (2017) 97:1055–64. 10.1002/jsfa.8062 27696415

[34] Ary-Sikorska E Fotschki B Fotschki J Wiczkowski W Jukiewicz J. Preparations from purple carrots containing anthocyanins improved intestine microbial activity, serum lipid profile and antioxidant status in rats. J Funct Foods. (2019) 60:103442. 10.1016/j.jff.2019.103442

[35] Patra AK Saxena J. Dietary phytochemicals as rumen modifiers: a review of the effects on microbial populations. Anton Leeuw. (2009) 96:363–75. 10.1007/s10482-009-9364-1 19582589

[36] Hosoda K Matsuo M Miyaji M Matsuyama H Maeda H Ohta H. Fermentative quality of purple rice (*Oryza sativa* L.) silage and its effects on digestibility, ruminal fermentation and oxidative status markers in sheep: a preliminary study. Grassl Sci. (2012) 58:1–9. 10.1111/j.1744-697X.2012.00256.x

[37] Tian XZ Paengkoum P Paengkoum S Thongpe S Ban C. Comparison of forage yield, silage fermentative quality, anthocyanin stability, antioxidant activity, and *in vitro* rumen fermentation of anthocyanin-rich purple corn (*Zea mays* L.) stover and sticky corn stover. J Integr Agr. (2018) 17:2082–95. 10.1016/S2095-3119(18)61970-7

[38] Song T Han O Park T Kim DW Yoon C Kim KJ. Anthocyanin stability and silage fermentation quality of colored barley. J Korean Soc Grassl Forage Sci. (2012) 32:335–42. 10.5333/KGFS.2012.32.4.335

[39] Messana JD Berchielli TT Arcuri PB Reis RA Canesin RC Ribeiro AF. Rumen fermentation and rumen microbes in nellore steers receiving diets with different lipid contents. Rev Bras Zootecn. (2013) 42:204–12. 10.1590/S1516-35982013000300008

[40] Chikwanha OC Emiliano R Opara UL Fawole OA Setati ME Voster M. Impact of dehydration on retention of bioactive profile and biological activities of different grape (*Vitis vinifera* L.) pomace varieties. Anim Feed Sci Tech. (2018) 244:116–27. 10.1016/j.anifeedsci.2018.08.006

[41] Wang Y Nan X Zhao Y Jiang L Xiong B. Effect of Supplementation of Inulin in Dietary on Lactation Performance, Rumen Fermentation, Ruminal Microbial Profile and Metabolites in Dairy Cows. Available online at: https://europepmc.org/article/PPR/PPR255102 (accessed December 18, 2020).

[42] Cardona F Andrés-Lacueva C Tulipani S Tinahones FJ Queipo-Ortuño MI. Benefits of polyphenols on gut microbiota and implications in human health. J Nutr Biochem. (2013) 24:1415–22. 10.1016/j.jnutbio.2013.05.001 23849454

[43] Igwe EO Charlton KE Probst YC Kent K Netzel ME. A systematic literature review of the effect of anthocyanins on gut microbiota populations. J Hum Nutr Diet. (2018) 32:53–62. 10.1111/jhn.12582 29984532

[44] Bryszak M Szumacher-Strabel M El-Sherbiny M Stochmal A Oleszek W Roj E. Effects of berry seed residues on ruminal fermentation, methane concentration, milk production, and fatty acid proportions in the rumen and milk of dairy cows. J Dairy Sci. (2019) 102:1257–73. 10.3168/jds.2018-15322 30580953

[45] Sun Z Yu Z Wang B. Perilla frutescens leaf alters the rumen microbial community of lactating dairy cows. Microorganisms. (2019) 7:562. 10.3390/microorganisms7110562 31766265 PMC6921060

[46] Chang LZ Zhong JC Xue SB Ding C Kang RM. Structure-activity relationships of anthocyanidin glycosylation. Mol Divers. (2014) 18:687–70. 10.1007/s11030-014-9520-z 24792223

[47] Pojer E Mattivi F Johnson D Stockley CS. The case for anthocyanin consumption to promote human health: a review. Compr Rev Food Sci Food Saf. (2013) 12:483–08. 10.1111/1541-4337.12024 33412667

[48] Cisowska A Wojnicz D Hendrich AB. Anthocyanins as antimicrobial agents of natural plant origin. Nat Prod Commun. (2011) 6:149–56. 10.1177/1934578X1100600136 21366068

[49] Gutierrez-Banuelos H Pinchak WE Min BR Carstens GE Anderson RC Tedeschi LO. Effects of feed-supplementation and hide-spray application of two sources of tannins on enteric and hide bacteria of feedlot cattle. J Environ Sci Heal B. (2011) 46:360–65. 10.1080/03601234.2011.559419 21547824

